# Developing and Evaluating a Health Literacy Training Model for Volunteer Elderly Caregivers to Prevent and Control NCDs in Thailand: An Action Research Study

**DOI:** 10.3390/nursrep16020068

**Published:** 2026-02-14

**Authors:** Phagapun Boontem, Jaruwan Phaitrakoon, Ninlapa Jirarattanawanna, Mayurachat Kanyamee, Siriporn Somboon, Kananit Sanghirun, Narunest Chulakarn

**Affiliations:** Faculty of Nursing, Srinakharinwirot University, Ongkharak 26120, Thailand; phagapun@g.swu.ac.th (P.B.); ninlapa@g.swu.ac.th (N.J.); mayurachat@g.swu.ac.th (M.K.); siriporns@g.swu.ac.th (S.S.); kananit@g.swu.ac.th (K.S.); narunest@g.swu.ac.th (N.C.)

**Keywords:** action research, community health volunteers, community-based intervention, diabetes, hypertension, health literacy framework, health promotion, older adults, Thailand

## Abstract

**Background/Objectives:** Limited health literacy among older adults with noncommunicable diseases (NCDs) remains a major challenge in community and primary-care settings. This action research aimed to develop and evaluate a community-based health literacy training model for volunteer caregivers for the elderly (VCEs) to support the prevention and control of diabetes and hypertension among older adults in the community. **Materials and Methods:** This study was conducted in a primary care-based community setting and comprised two phases: Phase 1 (model development) and Phase 2 (implementation and evaluation). The primary analytic sample consisted of 38 volunteer caregivers for the elderly, each providing home-based health education to one older adult (n = 38). The intervention combined structured health literacy education based on the K-shape framework (Knowledge, Comprehension, Thoughtful Inquiry, Decision-making, and Implementation) with SKT meditation/exercise. The program was delivered weekly over 8 weeks. Outcomes included health literacy (20-item scale) and disease prevention and control behaviors (12-item scale), assessed at baseline, immediately post-intervention, and 1 month after program completion. **Results:** Among VCEs, mean health literacy scores increased significantly from baseline to post-intervention and were further improved at 1-month follow-up (*p* < 0.001), indicating sustained gains in health literacy. Preventive behavior scores also increased significantly from baseline to post-intervention (*p* < 0.001); however, no additional improvement was observed at 1 month compared with immediately after the program (*p* > 0.05). The magnitude of improvement suggested a meaningful effect of the intervention on health literacy, while behavioral changes appeared to plateau after program completion. **Conclusions:** The community-based training model effectively and sustainably improved health literacy among volunteer caregivers for the elderly. Although preventive health behaviors improved immediately after the intervention, no further gains were observed at 1 month, suggesting that ongoing reinforcement may be required to sustain behavioral change. This model supports the role of community participation in primary care-based NCD prevention among older adults.

## 1. Introduction

The global population is ageing rapidly, with the number and proportion of individuals aged 60 years and older increasing across all regions. By 2030, one in six people worldwide, approximately 1.4 billion individuals, will be aged 60 years or older, up from 1 billion in 2020 [1]. The population aged 80 years and older is projected to triple in number between 2020 and 2050, reaching 426 million. Among older adults, the most prevalent conditions include hearing loss, cataracts, refractive errors, neck and back pain, osteoarthritis, chronic obstructive pulmonary disease (COPD), diabetes, depression, and dementia, with multimorbidity becoming increasingly common with advancing age [1]. The global burden of NCDs among the elderly is rising, yet health inequalities persist across age, sex, and socio-demographic index (SDI) levels.

From 1990 to 2021, there was a slight increase in the prevalence of Noncommunicable Diseases (NCDs) (e.g., diabetes mellitus, hypertension, and cardiovascular diseases) by 0.01%, whereas incidence, mortality, and Disability-Adjusted Life Years (DALYs) showed declines with rates of −0.04%, −0.99%, and −0.77%, respectively. Regions with a lower socio-demographic index exhibited a higher disease burden of NCDs, especially in terms of mortality and disability. Furthermore, the predictions for the NCD burden among the elderly from 2022 to 2050 indicated an incremental trend in prevalence. The elderly stage of life is characterized by physical decline and is often associated with underlying health conditions, particularly NCDs. These illnesses typically have a prolonged progression and tend to develop gradually over time. Common contributing factors to such conditions include poor dietary habits that fail to meet nutritional guidelines, smoking, persistent stress, and insufficient physical activity [2].

Thailand has transitioned into an aging society, evident from demographic statistics in 2024, when the elderly population reached 13,994,045 individuals. This figure accounts for 21.58% of the total population, with a dependency rate recorded at 28.93% [3]. A report published in 2022 by the Ministry of Public Health highlights data from the Health Data Center system as of 20 September 2022, showcasing records on 9,527,054 elderly individuals. Of this group, 7,501,688 individuals, or 78.74%, had participated in health assessments and screenings. The dominant health challenges among this demographic are chronic non-communicable diseases, predominantly hypertension, affecting 46.06%, followed by diabetes at 21.12%, and cerebrovascular conditions at 2.43% [4]. Based on data regarding NCDs in Bang O Subdistrict, Ban Na District, Nakhon Nayok Province, 84.52% of elderly individuals with such conditions were found to have high blood pressure, while 39.88% were diagnosed with diabetes. This information was reported by the Bang O Subdistrict Health Promotion Hospital in 2022.

Volunteer caregivers play a crucial role in providing care and support for older adults at the community level. However, these individuals often receive limited formal training and commonly lack adequate health literacy, particularly in relation to the prevention and management of NCDs. In many settings, there are no clearly defined or integrated protocols for training volunteer caregivers in NCD prevention, resulting in inconsistent practices and suboptimal health outcomes among older populations. This lack of standardized training frameworks underscores the need for targeted interventions aimed at strengthening health literacy among volunteer caregivers. A participatory, community-based approach that actively involves citizens is therefore essential to enhance knowledge, skills, and competencies related to NCD prevention, while fostering sustainable and contextually appropriate caregiving practices. Despite their central role, evidence-based models specifically designed to enhance health literacy among volunteer caregivers for NCD prevention and control remain limited, particularly within community and primary-care settings. However, most existing training programs for volunteer caregivers in Thailand primarily emphasize knowledge transfer and task-based caregiving, with limited attention to systematically developing health literacy skills such as critical appraisal, decision-making, and application in real-life caregiving contexts. Moreover, few programs are grounded in an explicit theoretical health literacy framework or evaluated using an action research approach within community and primary-care settings.

Elderly individuals suffering from NCDs need proper care to manage their conditions and avoid complications. However, although health promotion initiatives are underway, efforts to enhance health literacy among leaders remain unaddressed. This gap has led to inconsistent encouragement of self-learning, which directly affects overall health literacy. Health literacy encompasses an individual’s capability to obtain, comprehend, evaluate, and effectively apply health-related information, knowledge, and services. To enhance this, the Faculty of Nursing at Srinakharinwirot University, in collaboration with Bang O Subdistrict Health Promoting Hospital and the Bang O Subdistrict Administrative Organization in Thailand, has continuously worked on building the skills and competencies of Volunteer Caregivers for the Elderly (VCEs). VCEs are community-based elderly volunteers who support health promotion, disease prevention, and basic care for older adults, including activities related to noncommunicable disease prevention and control within the community. Unlike previous initiatives, this study proposes a structured health literacy training model specifically designed for Volunteer Caregivers for the Elderly (VCEs), integrating a theoretically grounded framework with participatory, community-based action research to address NCD prevention and control in real-world caregiving settings.

These efforts focus on enabling VCEs to provide care for the elderly in the Bang O Subdistrict community. Specifically, the aim is to empower VCEs to support elderly individuals with NCDs by emphasizing two key factors: proper nutrition and regular exercise. Elderly individuals with NCDs have consistently adjusted their behaviors to manage these chronic conditions. However, there is no definitive model in place to improve health literacy among elderly care volunteers, which is crucial for the prevention and control of NCDs. Moreover, efforts to enhance the health literacy of VCEs remain unaddressed. Consequently, there is a need to identify opportunities for ongoing improvement in health literacy development. In response to this need, the project titled “Promoting health literacy in volunteer caregivers for elderly care: An action research Model for Noncommunicable Disease Prevention and Control”. The research question was “What is an appropriate health literacy training model for Volunteer Caregivers for the Elderly (VCEs) to prevent and control noncommunicable diseases among the elderly?”

This initiative aims to increase health literacy among VCEs to ensure sustainable care, prevention, and control of NCDs in the elderly population. The health literacy organization principles of the Department of Health, Ministry of Public Health, guide the approach. These principles emphasize building health literacy through systematic development of individuals and organizations, focusing on enhancing the capabilities of organizational personnel and networks across all societal units. The goal is to create a health-literate Thai society [5]. Health literacy refers to the intellectual and social skills that guide motivation and an individual’s ability to access, understand, and use information to promote and maintain good health continuously [6]. The K-shape health literacy framework conceptualizes health literacy as a progressive and cyclical process, moving beyond functional knowledge toward higher-level cognitive and behavioral competencies. This project applies the K-shape health literacy promotion process, which comprises access, understanding, inquiring, deciding, and using. Within this framework, VCEs are supported to access and understand health information, actively inquire and critically appraise it, make informed decisions, and apply health knowledge in their caregiving roles to promote health and prevent noncommunicable diseases among older adults in the community [7,8]. This framework was selected because it aligns well with the roles of VCEs in community and primary-care contexts, emphasizing not only access and understanding of health information but also critical inquiry, shared decision-making, and practical application—competencies that are often underrepresented in conventional health education models. In this study, each component of the K-shape framework was operationalized through specific intervention activities, including structured health education sessions (access and understanding), guided group discussions and home-based questioning (inquiring), case-based decision-making exercises (deciding), and supervised caregiving practice incorporating lifestyle modification and SKT meditation/exercise (using).

These components serve as the framework for developing a model to enhance health literacy among VCEs, enabling them to effectively prevent and control NCDs in the elderly through community-empowered, participatory, and sustainable health care approaches that strengthen local capacity and support long-term health system sustainability within their own communities. By grounding the intervention in a clearly articulated health literacy framework and embedding it within a community-based action research design, this study contributes a theoretically informed and contextually relevant model for strengthening VCE capacity in NCD prevention and control among older adults in Thailand.

## 2. Materials and Methods

This study employed an action research design, which is appropriate for developing and implementing a health literacy training model through active participation of stakeholders and continuous reflection and improvement within a real community context. The study was reviewed and approved by the Research Ethics Committee of Srinakharinwirot University, Thailand (Approval No. SWUEC-672477, dated 2 December 2024). All participants received clear information regarding the study objectives and procedures, and written informed consent was obtained before participation.

### 2.1. Population and Sample

The sample size was determined using purposive sampling, which is appropriate for action research focusing on model development and implementation within a specific community context. The study was conducted in two phases as follows:

#### 2.1.1. Phase 1: Preparation

Qualitative target groups were selected using purposive sampling as follows:

Primary healthcare team (n = 24), comprising:

-10 VCEs

-10 community leaders, including elderly leaders, village health volunteers, and volunteer group leaders involved in community health promotion and volunteer activities, 

-3 nurse practitioners; and

-1 public health academic. 

Phase 1 represented the planning and co-design cycle of the action research process. Key stakeholders, including VCEs, community leaders, nurse practitioners, and public health academics, collaboratively identified priority problems in elderly care, gaps in health literacy competencies, and feasible strategies for NCD prevention and control within the community context.

#### 2.1.2. Phase 2: Implementation

Quantitative sample group: Select the sample group by specific method (Purposive sampling) as follows:

(2.1) The primary study participants consist of 38 VCEs from Villages No. 3, 7, 8, 9, and 10 in Bang O Subdistrict, Ongkharak District, Nakhon Nayok Province, Thailand. 

Inclusion criteria were as follows:

1. Experience in elderly care;
2. Age 18–80 years;
3. Ownership of a smartphone and ability to use it;
4. Ability to read and write Thai; and
5. Willingness to participate in the study.

Exclusion criteria were as follows:

1. No direct involvement in elderly care activities;
2. Cognitive impairment or severe physical or mental conditions that could limit participation in training activities or data collection; and
3. Lack of access to, or inability to use, a smartphone during the study period.

(2.2) The elderly participants were 38 persons (≥60 years) with non-communicable diseases, specifically diabetes and/or hypertension, residing in Villages No. 3, 7, 8, 9, and 10 in Bang O Subdistrict, Ongkharak District, Nakhon Nayok Province. All participants must be able to read and write Thai and provide voluntary consent to participate.

The study included two distinct participant groups: volunteer caregiver elders (VCEs) (*n* = 38) and elderly participants aged ≥60 years with non-communicable diseases (*n* = 38). These groups were recruited at a 1:1 ratio for the study.

Although minor variations in implementation occurred across villages due to contextual factors, these adaptations were planned as context-sensitive adjustments rather than separate study arms, and the core intervention components and evaluation procedures were applied consistently across all sites.

Potential confounding factors, including education level and prior caregiving experience, were addressed through the collection of baseline demographic and background data. Missing data were minimal and handled using complete-case analysis.

Data were collected between January and May 2025. Prior to participation, written informed consent was obtained from all participants.

**Figure 1:**
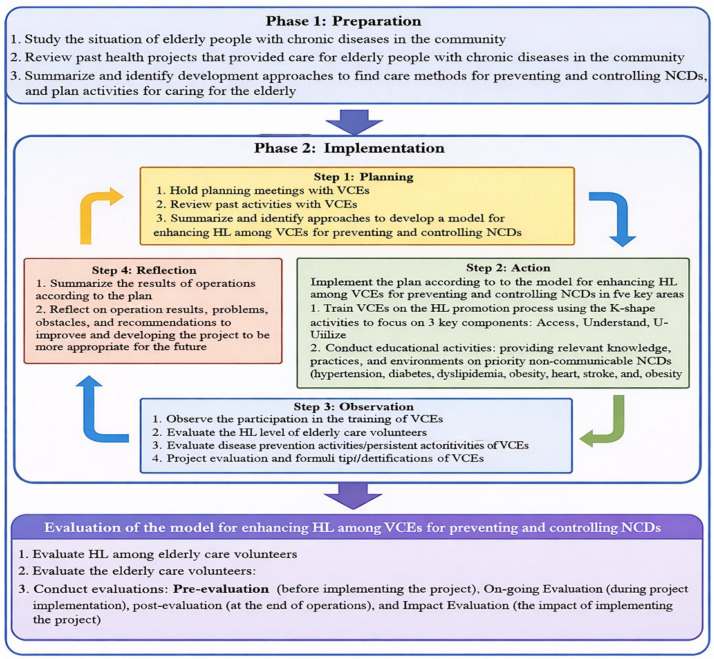
Steps of development of promoting HL in the VCE model for the prevention and control of NCDs in the elderly, Thailand.

The two-phase research process used to develop and implement a model for enhancing health literacy among VCEs for the prevention and control of NCDs, including preparation, implementation, and evaluation, as shown in Figure 1.

### 2.2. Research Tools

1. Focus Group Discussion Guide, used to develop a model for enhancing health literacy among VCEs for the prevention and control of NCDs within primary healthcare teams.

The FGD guide focused on the following aspects:

1. Identifying key problems in elderly care;
2. Priority areas for improvement; and
3. Feasible strategies for enhancing caregiving practices and health literacy among volunteer elderly caregivers.

Key outputs from the focus group discussions included:

1. Identification of priority health literacy domains;
2. Preferred learning methods;
3. Context-specific caregiving challenges; and
4. Recommendations for culturally appropriate training activities and materials.

2. Health Literacy Assessment Form [9]. The health literacy section comprises 20 items with 5 parts as follows:

1. Access to health information;
2. Understanding of health information;
3. Questioning and communication with health professionals;
4. Decision-making regarding health practices; and
5. Application of health information in daily life.

Each item is rated on a 5-point Likert scale ranging from 0 (unable to perform) to 4 (very easy to perform), classified into 4 levels:

4 = very easy to do, 

3 = easy to do, 

2 = difficult to do, 

1 = very difficult to do, 

0 = not done. 

Higher scores indicate higher levels of health literacy. Total and domain-specific scores were calculated to reflect participants’ health literacy levels. This form demonstrated acceptable internal consistency, with a Cronbach’s alpha coefficient of 0.81.

3. The Disease Prevention and Control Behavior Assessment Form [9]. Although the primary objective of the study was to enhance health literacy among VCEs, preventive behaviors were also assessed because health literacy is considered a key determinant of health-related behaviors. It measures the frequency of health behaviors among elderly care volunteers using a frequency-based rating scale corresponding to the number of weekly practice days. The instrument consisted of 12 items measuring participants’ routine health behaviors related to disease prevention and control. 

This has a 4-level scale as follows: 

4 = never practiced (never done); 

3 = sometimes practiced (1–2 days/week);

2 = often practiced (3–5 days/week); 

1 = always practiced (6–7 days/week). 

Responses were rated on a 4-point frequency scale, with higher scores reflecting better preventive behaviors. This form demonstrated acceptable internal consistency, with a Cronbach’s alpha coefficient of 0.78.

For ease of interpretation, preventive behavior scores were reverse-coded prior to analysis, such that higher scores indicated more frequent and desirable disease prevention and control behaviors.

4. Evaluation before the project implementation (Pre-evaluation), during the implementation (On-going Evaluation), evaluation of the project at the end of the implementation (Post-evaluation), and the impact of the project implementation (Impact Evaluation).

During the implementation phase, ongoing feedback from VCEs and supervising healthcare professionals was collected through informal discussions and ongoing evaluation, and minor adaptations to training activities and support strategies were made to better fit local contexts and participant needs.

### 2.3. Methods of Data Collection

1. Focus group discussions were conducted with primary healthcare team members using a semi-structured discussion guide. The discussions aimed to develop a health literacy enhancement model for elderly care volunteers and to plan activities for the prevention and control of NCDs in elderly care. 

Focus group discussions were conducted as follows:

1. Participants were organized into multiple focus groups, each facilitated by a trained researcher.
2. Discussions were audio-recorded with participant consent, transcribed verbatim, and supplemented with field notes.
3. Each session lasted approximately 60–90 min.
4. Transcripts were analyzed using thematic content analysis, with coding conducted independently by two researchers and discrepancies resolved through discussion.
5. Trustworthiness was enhanced through data triangulation across stakeholder groups and maintenance of an audit trail.

Qualitative findings directly informed the development of the conceptual model and intervention components.

Findings from Phase 1 focus group discussions were translated into the intervention design by aligning identified needs and suggestions with specific K-shape components, informing the selection of session content, training methods, and caregiving activities implemented during Phase 2.

2. VCEs implement elderly care activities following the K-shape health literacy framework. The focus was on preventing complications related to diabetes and hypertension, particularly those affecting the eyes, kidneys, and feet, as well as reducing the risk of stroke, and the initiatives also incorporated Somporn Kantharadussadee Traimchaisri (SKT) meditation therapy as a form of physical exercise. SKT Meditation Healing Exercise is a holistic mind–body practice developed by Professor Dr. Somporn Kantharadussadee Traimchaisri, integrating scientific principles with traditional meditation to promote physical and mental well-being. These activities included providing health information and education (access and understanding), encouraging discussion and question-asking about disease management (inquiring), supporting informed decision-making regarding preventive practices (deciding), and applying learned knowledge through routine care activities.

The intervention was delivered over a defined implementation period, consisting of weekly training and practice sessions facilitated by nurse practitioners and supervised by the research team. Standardized training materials and guidelines were used across all villages, with supervision ensuring consistency of implementation.

3. Health literacy and disease prevention and control behaviors among VCEs will be assessed at three time points using standardized instruments: before participating in the program, after participating in the program, and after 1 month of participating in the program. The content analysis was guided by the health literacy framework underpinning the intervention (K-shape health literacy promotion process), which informed the development of coding categories and interpretation of findings. These data collections were completed in person (on-site) by participants, with a member of the research team present to assist if needed.

### 2.4. Data Analysis

#### 2.4.1. Qualitative Data

Content analysis was used to analyze data from focus group discussions to identify themes and patterns related to the health literacy enhancement model development. Discussions were audio-recorded, transcribed verbatim, and analyzed iteratively through coding and theme development. Two researchers independently reviewed the transcripts and reached consensus through discussion, and data saturation was used to guide the completion of data collection.

#### 2.4.2. Quantitative Data

Descriptive Statistics

Means, standard deviations, frequencies, and percentages were calculated to describe participants’ characteristics, health literacy scores, and behavior scores at each time point.

Inferential Statistics

Inferential statistical analyses were conducted to examine changes in health literacy and disease prevention and control behavior outcomes. A dependent t-test was used to compare pre- and post-intervention scores. In addition, repeated measures analysis of variance (ANOVA) was employed to compare health literacy and disease prevention and control behavior scores across three time points: before participation in the program, immediately after participation, and one month after participation. Statistical significance was set at *p* < 0.05. All statistical analyses were performed using IBM SPSS Statistics, version 29.

## 3. Results

### 3.1. Qualitative Findings: Community Context and Perspectives of Health Professionals and Volunteers

#### 3.1.1. Problems of Elderly Care in the Community

Participants reported that most elderly individuals in the community were living with NCDs, particularly diabetes and hypertension, and frequently demonstrated inadequate self-care behaviors, such as irregular medication use and missed medical appointments.

“Elderly people in the community have chronic illnesses such as diabetes and high blood pressure. They don’t take their medication on time and don’t keep their doctor appointments. This can be seen from the amount of medication left over each time they visit the doctor.” (Health Volunteer No. 1)

However, some elderly individuals were still able to perform basic self-care activities with support from caregivers.

“Most elderly people are still able to take care of themselves and do some housework. Caregivers will take care of their diet, exercise, and medical appointments.” (VCE No. 3)

#### 3.1.2. Elderly Care Support in the Community

Community-Level Care Support

At the community level, VCEs served as the primary coordinators of elderly care, conducting regular home visits, monitoring health conditions, and facilitating access to healthcare services in collaboration with the Subdistrict Health Promotion Hospital.

“VCEs conduct monthly home visits and follow-up visits for patients with scheduled doctor appointments…” (Elderly Group Leader No. 2)

This finding reflects the integration of community-based volunteer care with the formal healthcare system.

Family-Level Care Support

At the family level, adult children played a crucial role in providing daily care and assisting elderly parents with healthcare navigation.

“Elderly people with children will have someone help take care of their meals, prepare for appointments, and bring their medications to see the doctor.” (VCE No. 1)

#### 3.1.3. Suggested Activities for Enhancing Health Literacy Among VCEs

K-shape conceptualizes health literacy as five interrelated competencies: access to health information, understanding of health-related messages, inquiry or questioning skills, decision-making based on available information, and application of health knowledge in real-life situations. The K-shape framework emphasizes the progressive development of practical health skills, making it particularly suitable for community-based interventions among VCEs. Importantly, suggested activities designed to enhance health literacy among VCEs may yield differential outcomes across these competencies, depending on the nature, intensity, and level of community engagement embedded within each activity.

Village-specific activities included the following:

- Villages 3 and 7: Health education focusing on NCD complications (diabetes- and hypertension-related complications).
- Villages 9 and 10: Integrated activities combining physical exercise and SKT meditation therapy.

Village health volunteers highlighted their role as intermediaries in disseminating health knowledge within the community.

“As Village Health Volunteers, we gain knowledge about various health issues. Once we gain this knowledge, we can share it with other elderly people.” (Village Health Volunteer No. 4)

These activities reflected the “application” component of the K-shape model, creating a multiplier effect for health knowledge dissemination.

**Figure 2:**
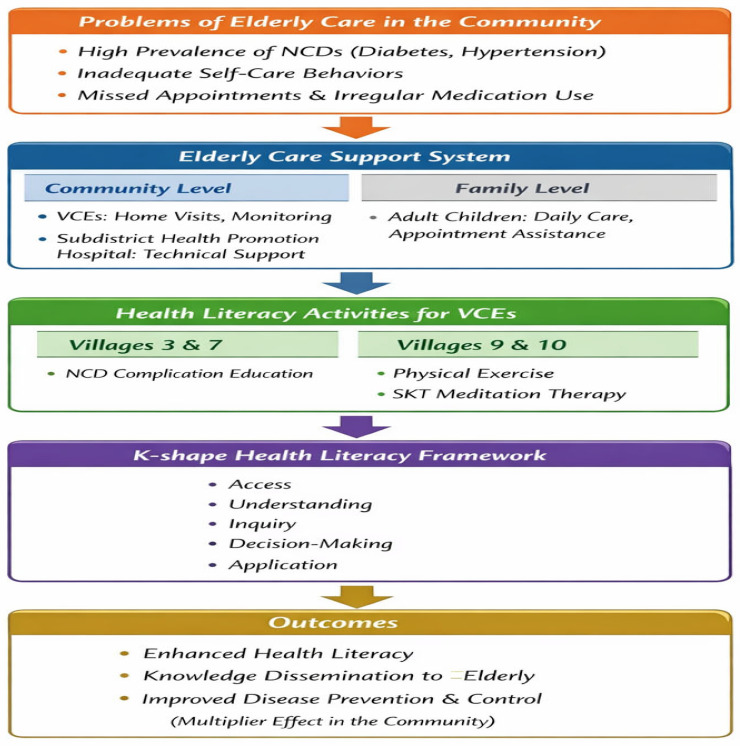
Conceptual model for enhancing health literacy among VCEs for NCD prevention and control, developed from qualitative findings.

Based on the qualitative findings, a conceptual model was developed to illustrate the process of enhancing health literacy among VCEs for the prevention and control of NCDs. The model integrates community-identified problems, existing elderly care support systems, health literacy activities, and the K-shape health literacy framework, as shown in Figure 2.

### 3.2. Quantitative Findings: Participant Demographics

The sample consisted of 38 VCEs, the majority of whom were female (86.8%). The mean age was 62.18 years (SD = 8.50). Most participants had completed primary education (52.6%), followed by secondary education (36.8%). The main sources of information on disease prevention and health practices were public health personnel (76.31%) and electronic media (31.57%).

Results of the Program for Developing a Model for Enhancing Health Literacy in Elderly Care Volunteers for the Prevention and Control of NCDs.

In this study, health literacy was conceptualized as a multidimensional construct encompassing five core components: access to health information, understanding of health-related messages, inquiry and communication with health professionals, decision-making regarding health practices, and application of health knowledge in daily life. These components were assessed using a standardized health literacy assessment instrument, and both domain-specific and overall health literacy scores were analyzed.

Table 1 shows the results indicate an improvement in both health literacy and disease prevention and control behaviors after the intervention, with scores remaining stable or slightly increased one month after program completion.

Table 2 shows that the mean health literacy score of the sample group after participating in the program was significantly higher than before participating in the program, t = 4.387, 95% CI 3.43, 9.31 (df = 37) *p*-value < 0.001, and the disease prevention and control behavior of the sample group after participating in the program was significantly higher than before participating in the program, t = −7.425 95% CI 7.979, 13.97 (df = 37) *p*-value < 0.001. The negative t-value reflects the direction of comparison and does not affect the interpretation of increased preventive behavior scores after reverse coding.

Repeated-measures ANOVA was conducted to examine changes in outcomes across three time points. Mauchly’s test indicated that the assumption of sphericity was violated for preventive behavior, χ^2^(2) = 11.62, *p* = 0.003. Therefore, the Huynh–Feldt correction was applied (ε = 0.812). The repeated-measures ANOVA revealed a significant main effect of time on preventive behavior, F(1.63, 60.12) = 43.17, *p* < 0.001, partial η^2^ = 0.54.

Table 3 presents changes in mean health literacy scores of the sample group across three time points: before participation, immediately after participation, and one month after participation in the program. Overall, health literacy scores differed significantly across the three time points (*p* < 0.001).

Post-intervention health literacy scores were significantly higher than baseline scores, indicating a clear improvement following participation in the program. Furthermore, health literacy scores at the one-month follow-up were significantly higher than immediate post-intervention scores, suggesting continued improvement and consolidation of health literacy over time.

Table 4 presents changes in disease prevention and control behavior scores of VCEs across three time points: before participation, immediately after participation, and one month after participation in the program. Disease prevention and control behavior scores increased significantly following participation in the program.

However, no further significant change was observed at the one-month follow-up, indicating that behavioral improvements were maintained but did not continue to increase over the short follow-up period. Thus, the prevention and control behaviors were sustained at the one-month follow-up, with no additional significant changes observed.

Table 5 summarizes the evaluation results across four stages of the project implementation, including pre-evaluation, ongoing evaluation, post-evaluation, and impact evaluation. Overall, the evaluation findings indicate progressive improvements across all stages of project implementation, with positive responses observed during implementation and sustained changes in health-related knowledge and behaviors at the post-implementation and follow-up stages.

## 4. Discussion

This study demonstrated that the training activity model designed to enhance health literacy among VCEs was effective in improving both health literacy and disease prevention and control behaviors related to NCDs among older adults. Importantly, the qualitative findings provide contextual insights that help explain how and why these improvements occurred within the community setting.

Qualitative data revealed that most older adults in the community were living with chronic NCDs, particularly diabetes and hypertension, and frequently exhibited inadequate self-care behaviors, such as irregular medication use and missed medical appointments. These findings highlight the complexity of NCD management among older adults and underscore the need for community-based support systems. At the same time, participants reported that many older adults retained the capacity for basic self-care when appropriate support from caregivers was available, suggesting that targeted interventions could effectively strengthen existing self-management abilities rather than replace them. This contextual understanding supports the observed quantitative improvements in health literacy and preventive behaviors following the intervention.

The role of community and family support emerged as a critical enabling factor in elderly care. At the community level, VCEs functioned as key coordinators of care, conducting home visits, monitoring health conditions, and facilitating access to healthcare services in collaboration with subdistrict health promotion hospitals. This integration of volunteer-led care with formal healthcare services provides a plausible explanation for the sustained behavioral outcomes observed at the one-month follow-up. At the family level, adult children played an essential role in supporting daily care, including dietary management, appointment preparation, and medication adherence. These findings suggest that the effectiveness of health literacy interventions may be enhanced when they align with existing family and community care structures.

The qualitative findings also clarify how the K-shape health literacy framework functioned in practice. Participants described activities that addressed multiple dimensions of health literacy, including access to information, understanding of health messages, inquiry and decision-making, and application of knowledge in real-life situations. Village-specific activities, such as education on NCD complications and integrated physical exercise and meditation programmes, reflect the adaptability of the K-shape model to local needs. VCE narratives highlighted their role as intermediaries in disseminating health knowledge, creating a multiplier effect that extended the benefits of the intervention beyond direct participants. This mechanism helps explain the magnitude of behavioral change observed quantitatively and supports the conceptualisation of health literacy as a dynamic and socially embedded process.

The stability of disease prevention and control behaviors at the one-month follow-up further supports the effectiveness of the intervention. This may be attributable to the village health volunteers’ roles as community leaders, in which consistent engagement in preventive behaviors is reinforced through social responsibility and ongoing interaction with community members. Similar observations have been reported in previous studies, indicating that even when health volunteers possess baseline knowledge, continuous support and structured programmes are necessary to sustain healthy behaviors and strengthen NCD prevention efforts within communities [10,11].

The continued improvement in health literacy at the one-month follow-up, in contrast to the stability of disease prevention and control behaviors, suggests that acquired health literacy skills may be more readily retained than behavior change. While knowledge and skills can persist once developed, sustained behavioral change is often constrained by environmental factors, physical limitations of older adults, and reliance on caregivers. These findings indicate that additional reinforcement, such as booster sessions or caregiver-focused support, may be required to translate sustained health literacy gains into further behavioral improvement.

Consistent with existing literature, enhancing health literacy among VCEs appears to increase their capacity to engage effectively in outreach activities and health promotion. For example, a study conducted in Japan found that municipal health promotion volunteers with higher health literacy levels were more actively involved in family- and community-based outreach, thereby strengthening the delivery of primary healthcare services [12]. These findings suggest that educational interventions aimed at improving health literacy can facilitate volunteer performance and broaden the reach of community health initiatives.

The present findings also support recommendations that volunteer development programmes should be systematically planned according to volunteers’ educational needs and accompanied by appropriate management strategies to recruit, motivate, and retain volunteers [13]. Empowering VCEs through recognition and opportunities to demonstrate their societal contributions may further enhance motivation and long-term engagement. In line with Adachi et al. (2019), individuals with lower health literacy tend to show limited interest in health information, underscoring the importance of targeted educational support to stimulate engagement and improve literacy levels [14]. Similarly, Tan et al. (2020) reported low health literacy related to hypertension among both community members and village health volunteers, emphasising the need for continuous training to support effective disease management and follow-up [15].

From a broader perspective, the results are consistent with evidence indicating that community-level health literacy interventions can improve health knowledge, skills, and behaviors, ultimately contributing to improved health outcomes [16]. Comparable intervention studies have demonstrated significant post-intervention improvements in health literacy and preventive behaviors [17].

### 4.1. Policy and Practical Implications

The findings of this study have important implications for public health policy and practice, particularly within primary healthcare and community-based NCD prevention systems. Enhancing health literacy among VCEs may be translated into sustainable public health policies by integrating structured health literacy training into routine volunteer development programmes at local and provincial levels. The training activity model developed in this study may serve as a practical framework for community health organisations and local administrative bodies to strengthen volunteer capacity and standardise health promotion activities. In practical terms, primary-care units and community organisations can adopt this model by integrating structured health literacy activities into routine VCE training, supervision, and home-visit programmes. Minimal conditions for implementation and scale-up include trained facilitators, collaboration with local health services, and context-appropriate educational materials, without requiring substantial additional resources.

In practical terms, incorporating health literacy modules into ongoing volunteer training, supervision, and evaluation processes may enhance the continuity and quality of care for older adults with NCDs. Such integration has the potential to support sustainable programme implementation and contribute to long-term reductions in NCD-related risks in ageing communities.

At the policy level, integrating health literacy development for community volunteers into national and local NCD management strategies may strengthen primary healthcare systems in ageing societies. Such policies could enhance continuity of care for older adults with chronic conditions and support sustainable, community-based approaches to NCDs prevention and control.

### 4.2. Achievement of Objectives and Study Limitations

The study successfully achieved its objectives by demonstrating significant improvements in health literacy and disease prevention and control behaviors among VCEs following participation in the intervention. Nevertheless, several limitations should be acknowledged. The use of a relatively small sample size and purposive sampling may limit the representativeness of the findings. In addition, the relatively short follow-up period restricted the assessment of the long-term sustainability of behavioral changes.

Uncontrolled variables, such as differences in educational background, prior caregiving experience, and levels of community support, may also have influenced the outcomes. Furthermore, reliance on self-reported measures may have introduced response bias.

In addition, the absence of a control group limits causal inference, and reliance on self-reported outcomes may be subject to social desirability and Hawthorne effects. The short follow-up period and village-level tailoring of activities may also complicate attribution of effects and limit generalizability.

### 4.3. Generalisability and Future Research Directions

Although this study was conducted within a specific community context, the findings may be generalisable to other rural or semi-urban settings with similar community health volunteer systems. However, socio-cultural, organisational, and health system differences across regions may limit direct transferability.

Future research should employ longitudinal designs to assess the sustainability of improvements in health literacy and behavioral outcomes over extended periods. Studies involving larger sample sizes, multiple study sites, and controlled or randomised designs are recommended to strengthen the evidence base and further inform policy development and programme implementation for NCDs prevention among older populations.

## 5. Conclusions

The training activity model developed in this study is an original approach to promote health literacy among elderly care volunteers in the prevention and control of NCDs. The model proved effective in significantly improving health literacy scores (*p* < 0.001). Although disease prevention and control behaviors were not fully sustained after one month (*p* > 0.05), the findings highlight the importance of continuing the programme over time to reinforce and maintain preventive behaviors. Although the intervention primarily enhanced health literacy among VCEs, related preventive behaviors were maintained at the same level at the one-month follow-up.

## Figures and Tables

**Table 1:** Mean and standard deviation of health literacy and disease prevention and control behaviors of elderly care volunteers (n = 38).

Health Literacy and Disease Prevention Behaviors	Mean (X)	Standard Deviation (S.D)
Health literacy		
-Before participating in the program	59.55	7.92
-After participating in the program	65.92	6.74
-After 1 month of participating in the program	71.05	8.81
Disease prevention and control behavior		
-Before participating in the program	20.29	9.35
-After participating in the program	31.26	5.39
-After 1 month of participating in the program	31.66	5.09

**Table 2:** Mean scores of health literacy and preventive behaviors of VCEs before and after participating in the program (n = 38).

Outcome Variable	Before Program (Mean ± SD)	After Program Mean ± SD	Mean Difference (95% CI)	t	df	*p*-Value
**Health** **literacy**	59.55 ± 7.92	65.92 ± 6.74	6.37 (3.43, 9.31)	4.387	37	<0.001 *
**Preventive behavior**	20.29 ± 9.35	31.26 ± 5.39	10.97 (7.98, 13.97)	7.425	37	<0.001 *

* p-value < 0.001 (Paired *t*-test).

**Table 3:** Mean health literacy scores of VCEs before, after, and after 1 month of participating in the program (n = 38).

Pairwise Comparisons						
(I) Time	(J) Time	Mean Difference (J − I)	Std. Error	Sig. *	95% Confidence Interval for Difference	
					Lower Bound	Upper Bound
1	2	6.368 *	1.452	0.000	3.427	9.310
	3	11.500 *	2.036	0.000	7.376	15.624
2	1	−6.368 *	1.452	0.000	−9.310	−3.427
	3	5.132 *	1.600	0.003	1.889	8.374
3	1	−11.500 *	2.036	0.000	−15.624	−7.376
	2	−5.132 *	1.600	0.003	−8.374	−1.889

* p-value < 0.01 (Repeated ANOVA measure). 1 = Before participating in the program, 2 = After participating in the program, and 3 = After 1 month of participating in the program.

**Table 4:** Mean scores of disease prevention and control behaviors of elderly care volunteers before, after, and after 1 month participating in the program (n = 38).

Pairwise Comparisons						
(I) Time	(J) Time	Mean Difference (J − I)	Std. Error	Sig. *	95% Confidence Interval for Difference	
					Lower Bound	Upper Bound
1	2	10.974 *	1.478	0.000	7.979	13.968
	3	11.368 *	1.628	0.000	8.070	14.667
2	1	−10.974 *	1.478	0.000	−13.968	−7.979
	3	0.395	0.976	0.688	−2.372	1.583
3	1	−11.368 *	1.628	0.000	−14.667	−8.070
	2	−0.395	0.976	0.688	−2.372	−1.583

* p-value < 0.01 (Repeated ANOVA measure). 1 = Before participating in the program, 2 = After participating in the program, and 3 = After 1 month of participating in the program.

**Table 5:** Results of the Evaluation Before the Project Implementation (Pre-Evaluation), During the Project Implementation (On-Going Evaluation), After the Project Implementation (Post-Evaluation), and the Impact of the Project Implementation (Impact Evaluation).

Evaluation	Key Findings
Pre-evaluation	Community stakeholders demonstrated a high level of cooperation and readiness to participate in the project.
On-going Evaluation	Participants actively engaged in the activities. However, behavioral change was gradual, and some participants required additional time to adopt new health-related practices.
Post-evaluation	Elderly participants showed positive responses to the activities, particularly those related to NCD prevention and management. SKT meditation was reported to be feasible for daily practice and contributed to improved well-being.
Post-evaluation 1 month	Participants demonstrated improved health awareness and reported applying skills related to information seeking, understanding, appraisal, and use in their daily health care practices.
Impact Evaluation	The project enhanced the capacity of volunteer caregivers to act as volunteer caregivers in the community, supporting elderly care activities and reducing caregiving burden within families.

## Data Availability

The data presented in this study are available on reasonable request from the corresponding author. The data cannot be publicly shared due to ethical and legal requirements, as the dataset contains sensitive personal health information and confidentiality must be protected.

## Public Involvement Statement

No public involvement in any aspect of this research.

## Guidelines and Standards Statement

This manuscript was drafted against the DOPHL (Development of the Promoting Health Literacy) for Action Research.

## Abbreviations

The following abbreviations are used in this manuscript:

VCEs	Volunteer Caregivers for the Elderly
NCDs	Non-communicable Diseases
SKT	Somporn Kantaradusdi-Triamchaisri
HL	Health Literacy
